# Supplementary material for: Purification of a Fc-Fusion Protein with [Bathophenathroline:metal] Complexes
Source: Antibodies (Basel). 2025 Jan 31;14(1):11. doi: 10.3390/antib14010011 (PMC11843901; doi:10.3390/antib14010011)
Supplement: Supplementary file 1 [file antibodies-14-00011-s001.zip › antibodies-3415101-supplementary.pdf]

Purification of an Fc-fusion protein with [bathophenathroline:metal] complexes

Supplementary Materials

Thisara Jayawickrama Withanage,[1] Ron Alcalay [2] , Olga Krichevsky [1] , Ellen Wachtel [3] , Ohad Mazor [2*] , and Guy Patchornik [1*]

[1] Department of Chemical Sciences, Ariel University, Ariel 4070000, Israel.

[2] Israel Institute for Biological Research, Ness Ziona 7410001, Israel.

[3] Faculty of Chemistry, Weizmann Institute of Science, Rehovot 7610001, Israel.

*Corresponding authors emails: ohadm@iibr.gov.il (O.M.); guyp@ariel.ac.il (G.P.)

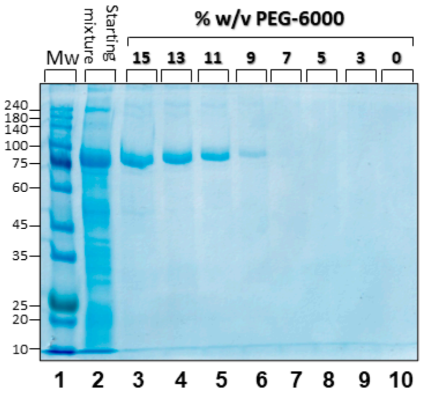

Figure S1: Purification process efficiency as a function of PEG-6000 concentration. Lane 1: Molecular weight markers; lane 2: Total amount of impure recombinant human AChE-Fc added to each purification trial; lanes 3-10: decreasing concentrations of PEG-6000 added to a constant amount of [$(batho)_3$:Zn^{2+}] as described in the Methods section, followed by extraction at 10°C during 30 minutes in the presence of 100 mM Na citrate (pH 7), 250 mM NaCl. The gel is Coomassie stained.

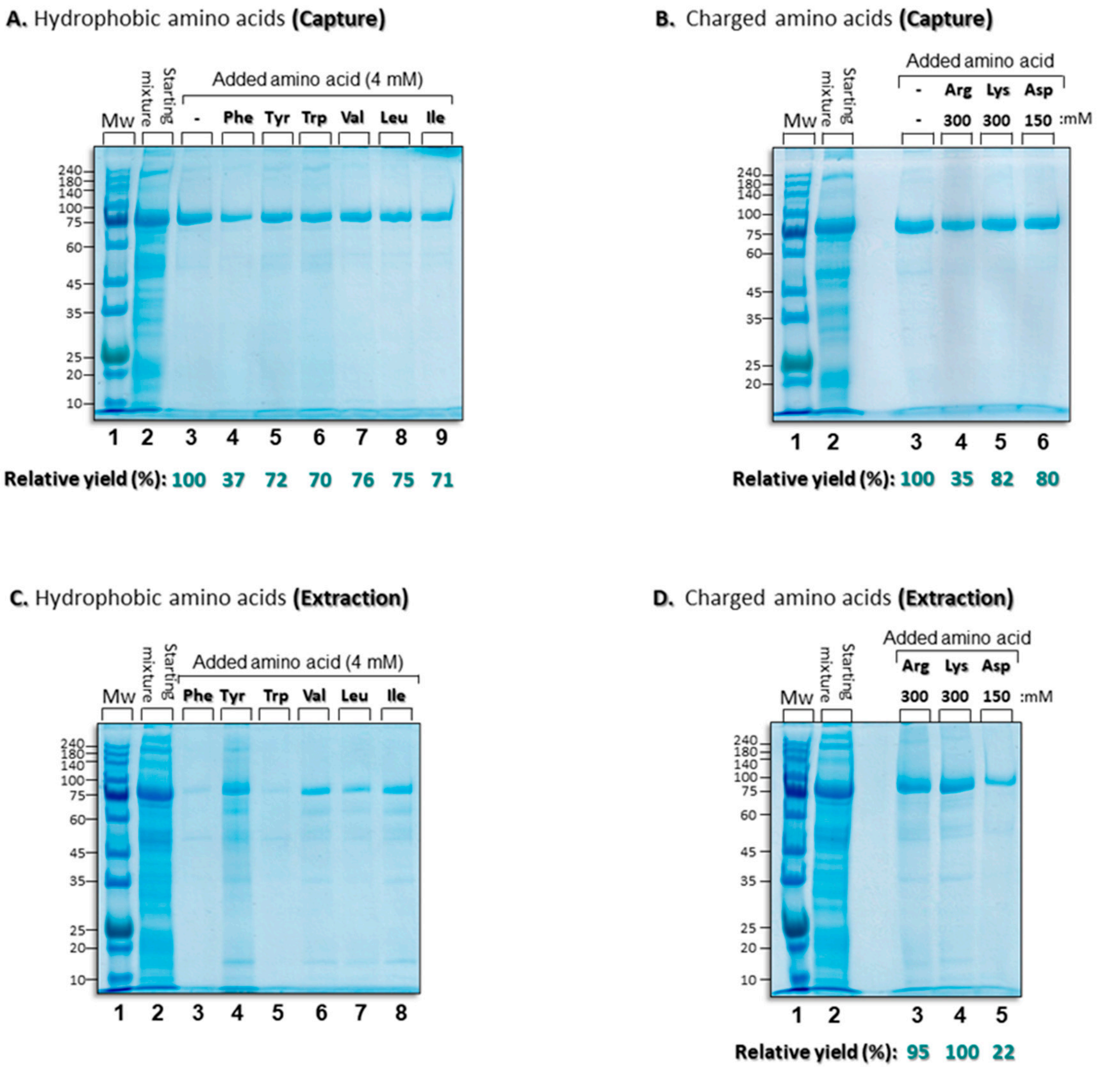

Figure S2: SDS-PAGE (with β-mercaptoethanol) analysis that focused on characterizing the dominant interactions between AChE-Fc and the [(batho)$_3$:Zn^{2+}] complex during the capture (**A-B**) and extraction (**C-D**) steps in the presence of 12% w/v PEG-6000 (as described in the Methods section). **A.** Lane 1: Molecular weight markers; Lane 2: Total amount of recombinant human AChE-Fc plus impurities added to each purification trial; Lanes 3-9: Recovered AChE-Fc following extraction with 0.1M Na citrate (pH 7), 250mM NaCl at 10°C for 30 minutes (lane 3) or in the presence of hydrophobic amino acids, pH7 added during the capturing-step **B**: As in **A**, but in the presence of hydrophilic amino acids. **C-D.** As in **A-B**, but when amino acid monomers are only added at the extraction step. The amino acids are first brought to pH 7, and then mixed with the pellet containing the [(batho)$_3$:Zn^{2+}] complex and the fusion protein. Relative process yields in panels **A**, **B** and **D** are indicated below the gel lane numbers and were quantitated using ImageJ (NIH). Gels are Coomassie stained.

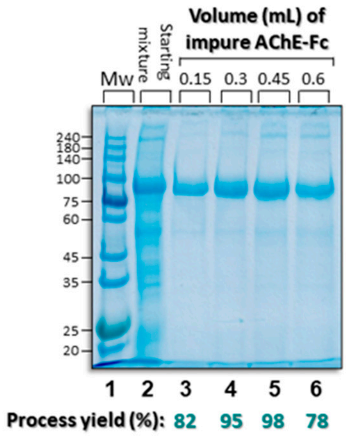

Figure S3: SDS-PAGE (with β-mercaptoethanol) gel for estimating the binding capacity of the [(batho)$_3$:Zn^{2+}] aromatic complex. Lane 1: Molecular weight markers; lane 2: Human AChE-Fc plus impurities (starting mixture); lanes 3-6: Recovered human AChE-Fc from indicated volumes using a constant amount of the [(batho)$_3$:Zn^{2+}] complex used for purification of the target from 150 µL as described in the Methods section. Process yields are indicated below the gel lane numbers and were quantitated using ImageJ (NIH) program. The gel is Coomassie stained.
